# Detection, characterization, and antibiogram of extended-spectrum beta-lactamase *Escherichia coli* isolated from bovine milk samples in West Bengal, India

**DOI:** 10.14202/vetworld.2018.1423-1427

**Published:** 2018-10-16

**Authors:** Kunal Batabyal, Abhiroop Banerjee, Susmita Pal, Samir Dey, Siddhartha Narayan Joardar, Indranil Samanta, Devi Prasad Isore, Abhishek Dharm Singh

**Affiliations:** 1 Department of Veterinary Microbiology, Faculty of Veterinary and Animal Sciences, West Bengal University of Animal and Fishery Sciences, Kolkata, West Bengal, India; 2 Department of Veterinary Public Health, F/VAS, West Bengal University of Animal and Fishery Sciences, Kolkata, West Bengal, India

**Keywords:** antibiogram, *bla*_CTX-M_, bovine milk, extended-spectrum beta-lactamase, *Escherichia coli*

## Abstract

**Background:**

Milk is considered as complete food and an important part of human diet throughout the world including India. Bacterial contamination of milk such as *Escherichia coli* due to unhygienic condition and poor udder health can cause infections, especially in infants and elders or in immunocompromised persons. Possession of antimicrobial resistance genes by commensal bacteria present in milk makes the issue more serious.

**Aim:**

The study was aimed to isolate and characterize extended-spectrum beta-lactamase (ESBL)-producing *E. coli* from milk samples collected from different parts of West Bengal, India, to assess the potential risk associated with the food.

**Materials and Methods:**

Around 182 milk samples were collected from apparently healthy cows reared by organized dairy farms in West Bengal. *E. coli* was isolated from collected samples as per standard methods followed by serotyping. The detection of ESBL-producing *E. coli* was done both phenotypically and genotypically by detecting the presence of *bla*_CTX-M_ gene. Antibiogram of the ESBL-positive isolates was done using common 12 antibiotics by disc diffusion method.

**Results:**

A total of 22 (12.1%) samples were found to be positive for *E. coli* in this study. Different serotypes such as O11, O20, O22, O34, O35, O128, O149, and UT were isolated from the collected samples. 12 (54.5%) *E. coli* strains showed the capability of producing ESBL, both phenotypically and genotypically with the presence of *bla*_CTX-M_ gene. Antibiogram of these ESBL-positive isolates revealed the drugs such as colistin (100%), levofloxacin (83.33%), and imipenem (66.67%) to be highly sensitive against this pathogen but drugs such as cefotaxime (100%), ceftazidime (91.67%), amoxicillin/clavulanic acid (83.33%), tetracycline (75.00%), and gentamicin (58.33%) to be very much resistant.

**Conclusion:**

More than 50% of the *E. coli* strains prevalent in the bovine milk samples were positive for ESBL production and are resistant to most of the common antimicrobials which may be alarming for human health.

## Introduction

India is one of the largest milk producing countries in the world with dairy industry playing an important role in the rural economy [1] generating huge self-employment. Bovine milk is generally considered to be a good source of protein and vitamins to human beings, particularly to the infants. However, due to faulty handling and storage of milk and poor management of the animal, milk may get spoiled due to rapid multiplication of bacteria due to milk’s high nutritive value [2]. *Escherichia coli* is one dreadful pathogen, especially the “enterohemorrhagic *E. coli*” strains, causing infection through milk which has a great effect on human health [3,4].

The prevalence of extended-spectrum beta-lactamase (ESBL)-producing *E. coli* is increasing in the globe including India. These pathogens pose a major challenge for the treatment of general infections and cause a problem with the extensive use of second- or third-generation antibiotics for the treatment of bacterial infections [5]. ESBL *E. coli* is mostly insensitive to lots of commonly used antibiotics causing an increase in the use of last-resort antimicrobial drugs (i.e., carbapenems) during treatment. Again, *E. coli* strains carrying the resistance genes can easily transfer those genes to other pathogens leading to the spread of drug resistance [6]. Hence, the presence of ESBL-producing *E. coli* in the food processing chain or in the food of our daily consumption which is possibly coming from these healthy farm animals is the fact which has to be appropriately studied.

The present study was aimed for the detection and characterization of ESBL-producing *E. coli* from raw milk samples (by detecting *bla*_CTX-M_ gene in the isolates) from different dairy farms followed by further characterization and to know their antibiotic resistance patterns *in vitro*.

## Materials and Methods

### Ethical approval

As per the guidelines of the Committee for the Purpose of Control and Supervision of Experiments on Animals, this study does not require any ethical approval from the University Animal Ethics Committee.

### Collection of samples

Bovine milk samples (n=182) were collected from different unorganized dairy farms of West Bengal aseptically in sterile plastic containers (Table-1) during the period of April-June, 2018. Milk samples were taken at 15 ml (approximately) in sterile plastic containers directly from the teats of the cows. The cows were selected on the basis of the history of illness/with decreased milk yield. The samples containers were kept in sample flask under ice and cooling pad cover and were transported through shortest route (for maximum 5-6 h approximately) to the Department of Veterinary Microbiology, Mohanpur, Nadia, for further study. All the collected samples were studied on the same day of receiving at the laboratory.

**Table-1:** Details of milk sample collection from different districts of West Bengal, India.

Name of the districts	Number of dairy farms covered	Total number collected samples
Purba Bardhaman	04	32
Paschim Bardhaman	05	47
Nadia	03	29
Hooghly	11	74
Total	23	182

### Isolation and characterization of E. coli

The collected milk samples were enriched adding sterile nutrient broth at 37°C for 6-8 h followed by streaking on to sterile MacConkey’s agar (HiMedia, India) plates. The plates were incubated aerobically at 37°C for 10-12 h. The tentative pinkish single colonies (i.e., lactose fermenting) were selected for selective isolation by further streaking on sterile Eosin Methylene Blue (EMB) agar (HiMedia, India) plates followed by 10-12 h incubation again at 37°C. The single colonies showing characteristic greenish “metallic sheen” were picked up and were stored using sterile nutrient agar (HiMedia, India) slants for further morphological study by Gram’s staining method and biochemical characterization with tests such as indole, methyl red, citrate utilization, Voges–Proskauer, catalase, and nitrate reduction [7,8]. All positive isolates showing typically to be *E. coli* were further confirmed for ESBL positivity later.

### Serotyping of the E. coli isolates

All positive *E. coli* isolates were sent to the National Salmonella and Escherichia Centre, Central Research Institute, Kasauli, Himachal Pradesh, India, for serotyping. All *E. coli* strains were subcultured in small sterile glass vials and were properly packed in hardboard box under cotton cover followed by sending to the NSEC, Kasauli, by registered post.

### Phenotypic detection of ESBL production in E. coli isolates

Phenotypic detection of the presence of ESBL in *E. coli* isolates was done *in vitro* by disc diffusion method [9] using both cefotaxime (30 µg) and ceftazidime disks (30 µg) with and without clavulanate (10 µg) as per the CLSI methods by Patel *et al*. [10]. A difference of >5 mm between the zone diameters of each disk and their respective clavulanate disk is measured to phenotypically confirm the ESBL production by the *E. coli* isolates under study [10].

### Molecular detection of ESBL production in E. coli isolates by polymerase chain reaction (PCR)

#### Bacterial culture lysate preparation

Selective *E. coli* strains were inoculated into nutrient broth (HiMedia, India) followed by 18 h incubation at 37°C. 1 ml of young broth culture of each sample was taken in a sterile 1.5 ml microcentrifuge tube (Tarsons, India) followed by centrifugation at 6000 rpm for 5 min [11]. The obtained pellet was washed 3 times with TE buffer and was suspended again in TE buffer (1 ml). The microcentrifuge tube with culture was then boiled in water for 10 min followed by chilling in ice. Again each tube was centrifuged at 5000 rpm for 5 min followed by removal of cell debris and the supernatant with crude DNA was collected and stored at −20°C for further use as a template in PCR [11].

#### Detection of bla_CTX-M_ gene (540 bp) in E. coli isolates

All phenotypically ESBL-positive *E. coli* isolates were considered for confirmation by PCR detection of the *bla*_CTX-M_ gene in them as per the protocol followed by Weill *et al*. [12] with slight modifications. All standard reagents and primers (GCC Biotech, India) were used in this process. Amplification reaction mixture containing 3 µl DNA templates, 50 pmol the primer set [540 bp] (forward_CTX-M_-F 5’-CAATGTGCAGCACCAGTAA-3’ and reverse_CTX-M_-R 5’-CGCGATATCATTGGTGGTG-3’), 1U GoTaq DNA polymerase (Promega, USA), 200 mM deoxynucleoside triphosphate, 10% dimethyl sulfoxide, and 2 mM MgCl_2_ was prepared in a 25 µl reaction mixture and used in PCR amplification conducted in a thermocycler (Eppendorf, Germany). The PCR amplification was done in the following cycle condition with an initial denaturation at 94°C for 10 min, followed by 30 cycles of denaturation at 94°C for 30 s, annealing at 53°C for 30 s, and elongation at 72°C for 60 s with a 10 min final extension period at 72°C. The amplified PCR products were loaded onto a 1.5% w/v agarose gel (SRL, India), with ethidium bromide (0.5 µg/ml) (SRL, India) followed by agarose gel electrophoresis and were visualized by gel documentation system (UVP, UK). One ESBL-producing *E. coli* strain (O2) which is the departmental isolate and one *Pseudomonas aeruginosa* (ATCC 27853) were used as positive and negative controls in PCR assays.

#### In vitro antibiotic sensitivity test of ESBL E. coli isolates

Antibiogram of the ESBL-positive *E. coli* isolates was performed using 12 antimicrobials, i.e., amikacin, amoxicillin/clavulanic acid, azithromycin, colistin, cotrimoxazole, cefotaxime, ceftazidime, gentamicin, imipenem, levofloxacin, piperacillin-tazobactam, and tetracycline by Kirby–Bauer disc diffusion method [9]. Young broth cultures of all the ESBL-positive isolates were produced for the test. Separate and sterile Mueller-Hinton agar (HiMedia, India) plates were used for uniform spreading of each broth culture using sterile L-spreader, and standard discs (HiMedia, India) were placed with sterile forceps. All the plates were incubated at 37°C for 10-12 h, and the results were interpreted by measuring the inhibition zone diameter and comparing those with the standard chart [10].

## Results

**Figure-1:**
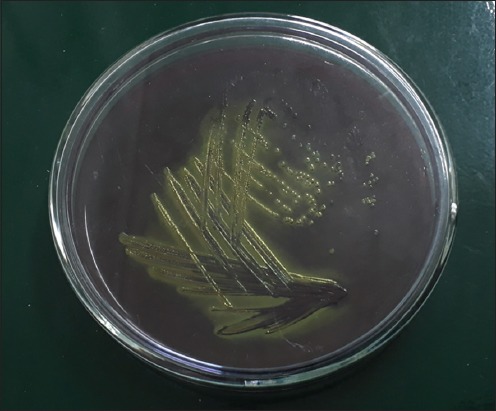
Characteristic “metallic sheen” produced by positive *Escherichia coli* isolated from bovine milk samples on Eosin Methylene Blue agar plate.

Out of 182 bovine milk samples tested, 22 (12.08%) samples were found to be identified as *E. coli* in this study. All the positive isolates showed typical characteristics during cultural, i.e., produced typical “metallic sheen” when grown on EMB agar plates (Figure-1) and morphological examinations (pink rods after Gram’s staining). All showed typical results during their biochemical characterization, i.e., positive to indole, methyl red, catalase, and nitrate reduction whereas negative to VP and citrate utilization.

Serotyping of all 22 *E. coli* isolates was done at the National Salmonella and Escherichia Centre, Central Research Institute, Kasauli, HP, India, to get the following 8 different serotypes, i.e., O11, O20, O22, O34, O35, O128, O149, and UT (Table-2).

**Table-2:** Frequency of *E. coli* serotypes isolated from bovine milk samples.

Sl. No.	*E. coli* serotypes	Frequency of prevalence (%)
1	O11	3 (13.63)
2	O20	7 (31.82)
3	O149	3 (13.63)
4	O22	5 (22.72)
5	O35	1 (4.55)
6	O34	1 (4.55)
7	O128	1 (4.55)
8	UT	1 (4.55)
Total		22 (100.00)

E. coli=Escherichia coli

**Figure-2:**
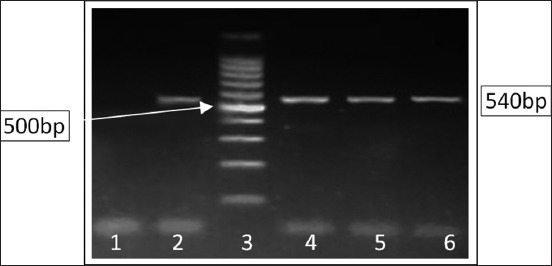
Polymerase chain reaction detection of *bla*_CTX-M_ gene (540 bp) in extended-spectrum beta-lactamase-producing *Escherichia coli* strains isolated from bovine milk samples in West Bengal, India. Lane 1: Negative control, Lane 2: Positive control, Lane 3: 100 bp ladder, Lanes 4-6: Test samples.

During ESBL detection, a total of 12 (54.54%) *E. coli* isolates were found to be phenotypically positive as ESBL producers by double disc method in this study. All phenotypically ESBL-positive *E. coli* isolates were detected to have the *bla*_CTX-M_ gene (540 bp) by PCR (Figure-2). The ESBL-producing strains belonged to O11[2 nos.], O20 [4 nos.], O22 [5 nos.], and O128 [1 no.]. Samples from Purba Bardhaman district showed the highest positivity in comparison to other districts (Table-3).

**Table-3:** District-wise Distribution of *E. coli* isolates with ESBL positivity.

Name of the Districts	Number of samples studied	Number of *E. coli* strains isolated (%)	ESBL positivity in *E. coli* strains
Purba Bardhaman	32	6 (18.75)	5
Paschim Bardhaman	47	7 (14.89)	4
Nadia	29	2 (6.90)	1
Hooghly	74	7 (9.46)	2
Total	182	22 (12.08)	12

*E. coli=Escherichia coli,* ESBL=Extended-spectrum beta-lactamase

*In vitro*, antibiotic sensitivity assay of the ESBL-positive *E. coli* isolates showed high-level resistance to cefotaxime, ceftazidime, amoxicillin-clavulanic acid, tetracycline, gentamicin, amikacin, etc. (with the range of 60-100%). Piperacillin-tazobactam (83.33%) was detected to be intermediately sensitive to these isolates (Table-4), and drugs such as colistin, levofloxacin, and imipenem were found to be sensitive against these pathogens.

**Table-4:** Antibiogram of 12 ESBL-producing *E. coli* strains isolated from bovine milk samples in West Bengal, India.

Sl. No.	Antimicrobials (Conc. in µg)	Isolates sensitive	Isolates intermediately sensitive	Isolates resistant
		
		n (%)	n (%)	n (%)
1.	Amikacin (30)	2 (16.67)	3 (25.00)	7 (58.33)
2.	Amoxicillin/Clavulanic acid (20/10)	2 (16.67)	0 (0)	10 (83.33)
3.	Colistin (10)	12 (100)	0 (0)	0 (0)
4.	Cotrimoxazole (25)	5 (41.67)	4 (33.33)	3 (25.00)
5.	Cefotaxime (30)	0 (0)	0 (0)	12 (100)
6.	Ceftazidime (30)	0 (0)	1 (8.33)	11 (91.67)
7.	Imipenem (10)	8 (66.67)	4 (33.33)	0 (0)
8.	Gentamicin (10)	0 (0)	5 (41.67)	7 (58.33)
9.	Levofloxacin (5)	10 (83.33)	2 (16.67)	0 (0)
10.	Pipercillin-Tazobactam (100/10)	2 (16.67)	10 (83.33)	0 (0)
11.	Azithromycin (30)	4 (33.33)	3 (25.00)	5 (41.67)
12.	Tetracycline (30)	2 (16.67)	1 (8.33)	9 (75.00)

ESBL=Extended-spectrum beta-lactamase, *E. coli=Escherichia coli*

## Discussion

Approximately 12% of the total milk samples screened were found to yield *E. coli* isolates in this study which were also supported by Kamaruzzaman [13], Badri *et al*. [14], Geser *et al*. [15], and Ali *et al*. [16] who also reported 12.22-13.7% *E. coli* positivity in the bovine milk samples during their study. All positive *E. coli* isolates showed typical cultural, morphological, and biochemical nature in this study which was also supported by Carter and Wise [7], Samanta [17], and Quinn *et al*. [8].

The serotypes reported by the National Salmonella and Escherichia Centre, Central Research Institute, Kasauli, were also supported by Osman *et al*. [18] who detected *E. coli* serogroups O26, O86, O111, and O127 from cattle milk in their study. Al-Zogibi *et al*. [19] reported the prevalence of *E. coli* serogroups, namely O22, O111, O113, and O172, from bovine milk samples in their study which also supports the current findings.

Approximately, 54.54% *E. coli* isolates were both phenotypically and genotypically positive to produce ESBL in this study which was also supported by Geser *et al*. [15] and Ibrahim *et al*. [20]. Kamaruzzaman [13] reported a high prevalence of ESBL-producing *E. coli* in milk (66.7%) followed by farm environment (27.8%) and cattle (5.5%) in his work. Ali *et al*. [16] reported 36 (23.53%) and Badri *et al*. [14] reported 29.3% ESBL-positive *E. coli* strains from bovine milk samples which may be of great concern as these pathogens may be carried out to the human consumers as well as calves leading to the spread of the antibiotic-resistant pathogens over human and animal population. Sharma *et al*. [21] also reported ESBL-positive *E. coli* serotypes in their study, matching the current findings.

The high level of antibiotic resistance as shown in this report was also reported earlier by Kamaruzzaman [13], Ibrahim *et al*. [20], and Hinthong *et al*. [22]. Ali *et al*. [16] also found resistance against drugs such as ampicillin (86.11%), amoxicillin-clavulanic acid (63.89%), cefotaxime (100%), ceftazidime (66.67%), tetracycline (72.22%), and gentamicin (61.11%) by ESBL *E. coli* pathogens in their study. Faruk *et al*. [23] reported that ampicillin, cefotaxime, ceftazidime, and cefuroxime (all 100%) and tetracycline (93.54%) were highly resistant but imipenem (100%) to be highly sensitive to the ESBL *E. coli* strains isolated from cattle in their study which almost matches with the current findings.

## Conclusion

The drug-resistant ESBL gene is significantly present in approximately 55% of the *E. coli* strains isolated from cattle milk samples which may be of great health concern for human beings. This drug resistance can easily be transferred between closely related pathogens *in vivo* which may result in risky and fatal health hazards due to unsuccessful treatment with common antimicrobials. Hence, proper care should be taken to combat these dreadful pathogens.

## Author’s Contributions

KB and SD designed the study. AB and ADS collected the samples. KB, AB, and SP carried out the experiment. SNJ, SD, and IS analyzed the data. DPI, KB, and SD drafted the article. SNJ and IS revised the article. All authors read and approved the final manuscript.
